# Identification of hub genes and immune infiltration in ulcerative colitis using bioinformatics

**DOI:** 10.1038/s41598-023-33292-y

**Published:** 2023-04-13

**Authors:** Weitao Hu, Taiyong Fang, Mingxuan Zhou, Xiaoqing Chen

**Affiliations:** 1 Department of Rheumatology, The Second Affiliated Hospital of Fujian Medical University, 34 North Zhongshan Road, Licheng District, Quanzhou, 362000 Fujian People’s Republic of China; 2 Department of Gastroenterology, The Second Affiliated Hospital of Fujian Medical University, Quanzhou, 362000 Fujian People’s Republic of China; 3 Department of General Practice, The Second Affiliated Hospital of Fujian Medical University, Quanzhou, 362000 Fujian People’s Republic of China

**Keywords:** Immunology, Gastroenterology, Rheumatology

## Abstract

Ulcerative colitis (UC) is a chronic inflammatory disease of the intestine, whose pathogenesis is not fully understood. Given that immune infiltration plays a key role in UC progression, our study aimed to assess the level of immune cells in UC intestinal mucosal tissues and identify potential immune-related genes. The GSE65114 UC dataset was downloaded from the Gene Expression Omnibus database. Differentially expressed genes (DEGs) between healthy and UC tissues were identified using the “limma” package in R, while their Gene Ontology (GO) and Kyoto Encyclopedia of Genes and Genomes (KEGG) pathways were determined with the clusterProfiler package. Protein–protein interaction network analysis and visualization were performed with STRING and Cytoscape. Immune cell infiltration was calculated with CIBERSORT. The relationship between hub genes and immune-infiltrated cells in UC was determined by Pearson correlation. A total of 206 DEGs were identified, of which 174 were upregulated and 32 downregulated. GO and KEGG functional classification indicated DEG enrichment in immune response pathways, including Toll-like receptor signaling, IL-17 signaling, and immune system process and chemokine signaling. 13 hub genes were identified. Infiltration matrix analysis of immune cells showed abundant plasma cells, memory B cells, resting CD4 memory T cells, γδ T cells, M0 and M1 macrophages, and neutrophils in UC intestinal tissues. Correlation analysis revealed 13 hub genes associated with immune-infiltrated cells in UC. 13 hub genes associated with immune-infiltrated cells in UC were identified; they included CXCL13, *CXCL10*,* CXCL9*,* CXCL8*,* CCL19*,* CTLA4*,* CCR1*,* CD69*,* CD163*, *IL7R*, *PECAM1*, *TLR8* and *TLR2*. These genes could potentially serve as markers for the diagnosis and treatment of UC.

## Introduction

Ulcerative colitis (UC) is a chronic inflammatory disease of the intestine, whose etiology remains poorly understood^1^. The diagnosis of UC is based on a combination of nonspecific symptoms, endoscopic findings, and histological features, which makes it sometimes difficult to discriminate UC from other diseases^2,3^. Although several drugs are available for the treatment of UC^4^, up to 15% of patients do not respond to drug therapy or have chronic colitis secondary to dysplasia, which requires surgery^5^. Therefore, there is an urgent need to better understand the pathogenesis of UC and identify more effective treatments.

Autoimmune mechanisms have long been hypothesized to be involved in the pathogenesis of UC^4^. Previous studies have suggested the presence of immune cells in the intestinal mucosa of UC patients^6^. Multiple environmental factors can interfere with the microbial ecosystem of the colon and determine how gut microbes interact with immune cells, thereby provoking an uncontrolled inflammatory response and exacerbating UC symptoms^7^. Infection or dysbiosis may disrupt the natural immune tolerance of genetically susceptible individuals, causing immune imbalance in the intestinal mucosa and further affecting the onset and progression of UC^8^. It has been shown that the development of new therapeutic approaches to modulate the gut microbiota through the administration of bacterial strains that produce probiotic immune metabolites and the addition of specific prebiotics and fecal microbiota transplants can treat UC patients requiring ileal pouch ano-anastomosis (IPAA)^9^. All these findings suggest a critical role of immune cells in the pathogenesis of UC. Therefore, understanding the pathogenesis of UC from the perspective of immune infiltration may hold the key for early treatment and prevention of UC-induced deterioration. In particular, it may provide a new approach for targeted immunotherapy of UC.

Bioinformatics can reveal the molecular mechanisms of disease through large-scale gene or protein expression profiling in diseased vs. healthy tissues. Expression data are especially useful to study dynamic regulation among multiple immune cells. CIBERSORT is a commonly used analytical tool in studies of tumor immunity, whereby information about cell subsets is derived from bulk gene expression data. The method, however, has not been applied widely to investigate non-tumor immunity or to analyze immune infiltration in UC. The present study aimed to identify potential UC biomarkers and assess the level of immune cells in intestinal mucosal tissues of UC patients using CIBERSORT. Furthermore, to identify immune-related genes suitable for the diagnosis and treatment of UC, the correlation between immune cells and hub genes was calculated.

## Materials and methods

### Microarray data

The Gene Expression Omnibus (GEO; www.ncbi.nlm.nih.gov/geo/)^10^ database was used to search gene microarray data of UC intestinal tissues. The GSE65114 dataset (Contributed by Balfe A, Lennon G, O’Connell R et al.: https://www.ncbi.nlm.nih.gov/geo/query/acc.cgi?acc=GSE65114), containing 12 healthy (control group) and 16 UC (experimental group) colon tissue samples was selected. Probes were converted to gene symbols according to the GPL16686 [HuGene-2_0-st] Affymetrix Human Gene 2.0 ST Array [transcript (gene) version] platform.

### Identification of differentially expressed genes (DEGs)

The gene expression matrix of the GSE65114 dataset was analyzed with the “limma” package in R to obtain DEGs between UC and healthy samples. Briefly, |log2 fold change (FC)| > 1 and *P* < 0.05 were set as the selection criteria for DEGs, with |log2FC| < 0 indicating downregulated genes and |log2FC| > 0 indicating upregulated genes when UC vs healthy individuals.

### Protein–protein interaction (PPI) network construction and module analysis

The PPI network was constructed using the Search Tool for the Retrieval of Interacting Genes (STRING; http://string-db.org) (version 11.5)^11^ online database, and the parameters were set as follows: meaning of network edges: confidence level; minimum required interaction score: medium confidence (0.400). Identification of functional interactions between proteins may provide insights into the mechanisms underlying disease development. The PPI network was drawn using Cytoscape (version 3.9.1), an open-source bioinformatics platform for visualizing molecular interactions^12^. The most significant module was identified with the Cytoscape plug-in Molecular Complex Detection (MCODE) (version 2.0), which is used to identify densely connected regions by clustering a given network based on its topology^13^. The criteria for selection were as follows: MCODE score > 5, degree cut-off = 2, node score cut-off = 0.2, maximum depth = 100, and k-score = 2. Using the cytoHubba plugin, the overlap of the top 20 genes based on algorithms such as MCC, maximum neighborhood component (MNC), Closeness, Degree, and edge percolated component (EPC) algorithms were identified as hub genes of UC.

### Pathway enrichment analyses of DEGs

The clusterProfiler package in R was used to identify the Gene Ontology (GO) and Kyoto Encyclopedia of Genes and Genomes (KEGG)^14–16^ pathways characterizing DEGs, as well as to explore their potential biological processes, cellular components, molecular functions, and important signaling pathways. *P* < 0.05 and false detection rate < 0.1 were considered statistically significant.

### Evaluation of subtype distribution among immune-infiltrated cells

CIBERSORT was shown to transform a normalized gene expression matrix into the composition of 22 immune cell types based on a deconvolution algorithm^17^. Here, CIBERSORT was employed to calculate the composition of immune cells in UC and healthy samples. The algorithm employed the LM22 signature and 1000 permutations. Given *P* < 0.05, 16 UC and 12 healthy samples were selected for further analysis.

### Correlation and differential analysis of immune-infiltrated cells

To assess the correlation among various immune cells, the Pearson correlation coefficient was calculated from sample data screened by CIBERSORT with *P* < 0.05. The rank-sum test was used to compare the UC and control groups.

### Correlation between hub genes and immune-infiltrated cells in UC

The gene expression profile and the immune infiltrating cell profile of the CIBERSORT-analyzed GSE65114 dataset were subjected to Pearson correlation matrix analysis. Using Pearson correlation coefficient (r) > 0.6 and *P* < 0.05, the correlation between hub genes and immune-infiltrated cells was determined.

## Results

### Identification of DEGs in UC

**Figure 1:**
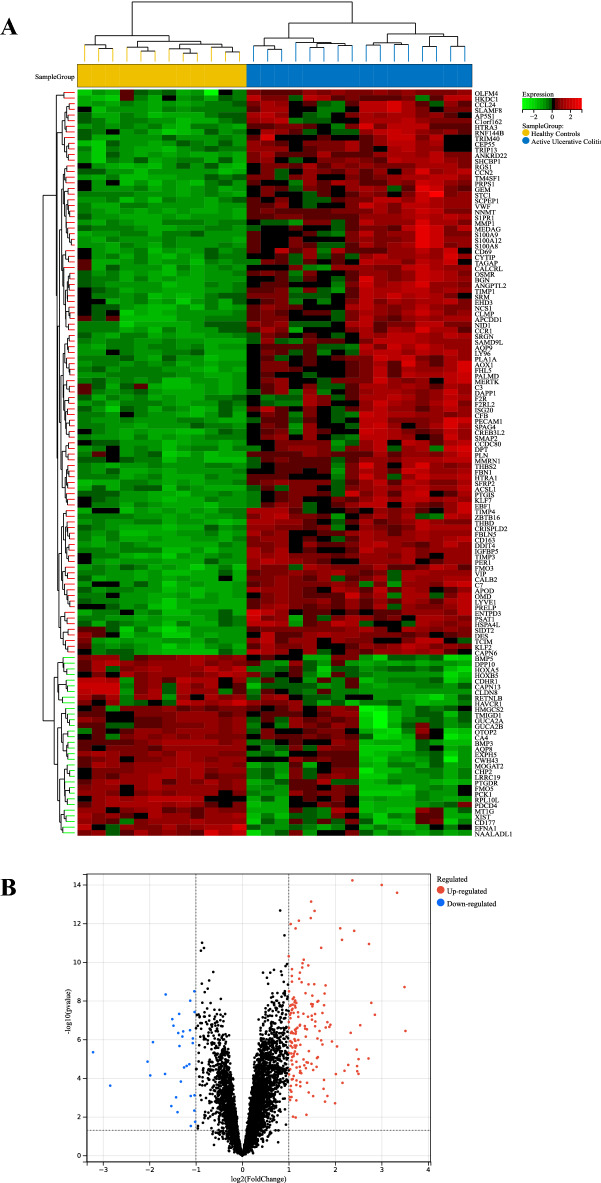
Differential gene expression in colonic mucosa tissue of UC patients. (**A**) Heat map of DEGs. Green corresponds to lower gene expression and red to higher gene expression. (**B**) Volcano plot of differentially expressed genes. |Log2FC| ≥ l and *P* < 0.05 were chosen as filtering conditions. Blue dots represent significantly downregulated genes, red dots represent significantly upregulated genes, and black dots denote genes not showing any significant difference.

The GSE65114 dataset containing gene expression profiles of 12 healthy and 16 UC active colonic mucosa tissue samples was retrieved from the GEO database. After standardization of microarray results, 206 DEGs were identified; they included 174 upregulated and 32 downregulated genes (Fig. 1).

### PPI network construction, module analysis, and hub gene identification

**Figure 2:**
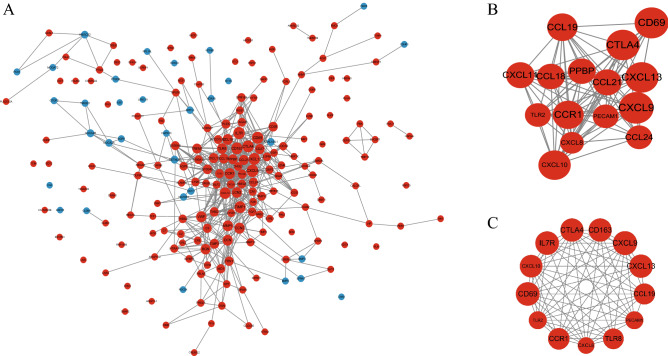
PPI network construction and module analysis. (**A**) PPI network of DEGs. The network consists of 206 nodes (39 isolates) and 603 edges, and network Diameter is 12, network Sparseness is 0.049, and Avg clustering coefficient is 0.424. Upregulated genes are marked in red and downregulated genes are marked in blue. The size of nodes represents the difference in expression; the larger is the size, the more significant is the *P* value. (**B**) Cytoscape-based identification of the densest connected regions (15 nodes and 87 edges) in the PPI network. (**C**) Hub genes identified in the densest connected regions. A darker red hue indicates a higher score.

PPI analysis of DEGs was based on the STRING database and results were visualized using Cytoscape (Fig. 2A). The MCODE plug-in identified the most densely connected regions (15 nodes and 87 edges) in the PPI network (Fig. 2B). The complete Cytoscape’s SIF file about the PPI network was in the Supplementary file. With the 5 algorithms of the Cytoscape plugin cytoHubba, we calculated the top 20 genes (Table 1). Subsequently, the top 13 intersecting genes analyzed based on these 5 algorithms were selected as the hub genes of UC, which included *CCL19*,* CCR1*,* CD163*,* CD69*,* CTLA4*,* CXCL10*,* CXCL13*,* CXCL8*,* CXCL9*,* IL7R*,* PECAM1*,* TLR2* and *TLR8* (Fig. 2C). Notably, in our study, all these hub genes were upregulated in patients with UC.

**Table 1:** The top 20 hub genes rank in cytoHubba.

MCC	MNC	Degree	EPC	Closeness	Overlap
CXCL8	DCN	DCN	DCN	CXCL8	
CTLA4	CXCL8	CXCL8	CXCL8	CTLA4	
IL7R	CTLA4	CTLA4	CTLA4	MMP1	
CCL21	MMP1	MMP1	MMP1	IL7R	
CXCL13	IL7R	IL7R	IL7R	CXCL13	**CCL19**
CCR1	CXCL13	CXCL13	CXCL13	CCL21	**CCR1**
TLR8	TLR8	TLR8	TLR8	TLR8	**CD163**
CXCL9	CCR1	CCR1	CCR1	CCR1	**CD69**
CCL19	VWF	VWF	VWF	VWF	**CTLA4**
CCL24	CXCL9	CXCL9	CXCL9	CXCL9	**CXCL10**
TLR2	CCN2	CCN2	CCN2	CCL19	**CXCL13**
CCL18	CCL19	CCL19	CCL19	TLR2	**CXCL8**
CD163	BGN	BGN	BGN	CD163	**CXCL9**
CD27	TLR2	TLR2	TLR2	TIMP1	**IL7R**
TREM1	CD163	CD163	CD163	TREM1	**PECAM1**
CXCL10	TIMP1	TIMP1	TIMP1	CXCL10	**TLR2**
PECAM1	CXCL10	CXCL10	CXCL10	PECAM1	**TLR8**
PPBP	PECAM1	PECAM1	PECAM1	PPBP	
CD69	LCN2	LCN2	CD69	CD69	
CXCL11	CD69	CD69	CXCL11	CXCL11	

Significant values are in bold.

### KEGG and GO enrichment in DEGs

**Figure 3:**
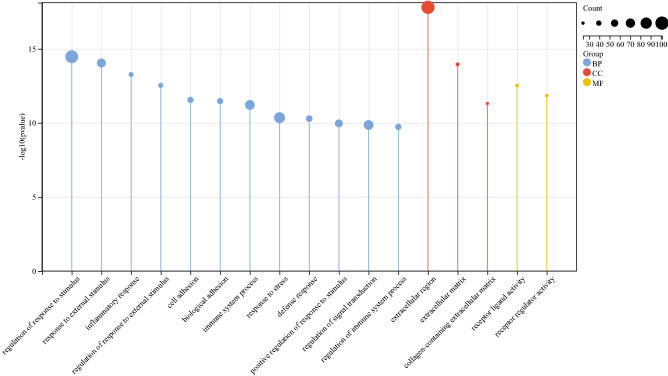
GO enrichment analysis of DEGs related to UC. A darker color and a larger bubble denote a more significant difference.

**Figure 4:**
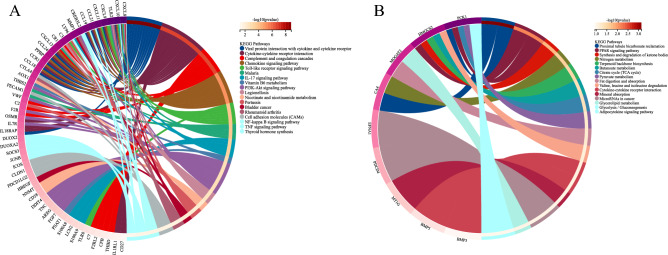
KEGG enrichment analysis of DEGs related to UC. (**A**) Upregulated DEGs. (**B**) Downregulated DEGs. The genes are linked to their assigned pathway terms via colored ribbons and are ordered according to the observed log10 *P*-value, which is displayed in descending intensity of red squares next to the selected genes.

To predict the biological function of DEGs, we performed functional enrichment analysis. GO analysis revealed that the DEGs were enriched mainly in extracellular region, regulation of response to stimulus, inflammatory response, immune system process and defense response (Fig. 3). KEGG pathway analysis indicated that upregulated genes were significantly enriched in viral protein interaction with cytokine and cytokine receptor, cytokine-cytokine receptor interaction, complement and coagulation cascades, chemokine signaling pathway, Toll-like receptor signaling pathway, and IL-17 signaling pathway (Fig. 4A); whereas downregulated genes were enriched mainly in proximal tubule bicarbonate reclamation and PPAR signaling pathway (Fig. 4B).

### Distribution of immune-infiltrated cells

**Figure 5:**
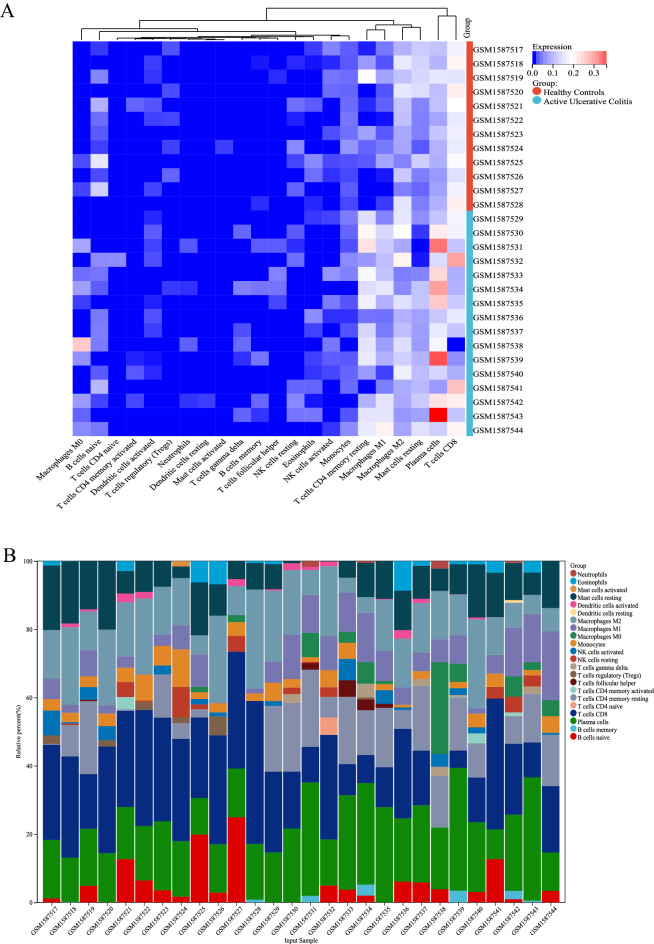
Infiltration of immune-associated cells in healthy and UC samples. (**A**) Immune cell content in each sample. (**B**) Relative percentage of 22 subpopulations of immune cells in 28 samples from the GSE65114 dataset.

The microarray was screened using the CIBERSORT inverse convolution method with *P* < 0.05, resulting in 12 healthy intestinal tissues at the top of the heat map and 16 UC intestinal tissue groups at the bottom. Plasma cells, memory B cells, resting CD4 memory T cells, γδ T cells, M0 macrophages, M1 macrophages, and neutrophils were all more abundant in colonic mucosal tissues of patients with active UC than in healthy controls (Fig. 5A). Figure 5B details the distribution of 22 immune cells in each sample.

### Correlation and differential analysis of immune-infiltrated cells

**Figure 6:**
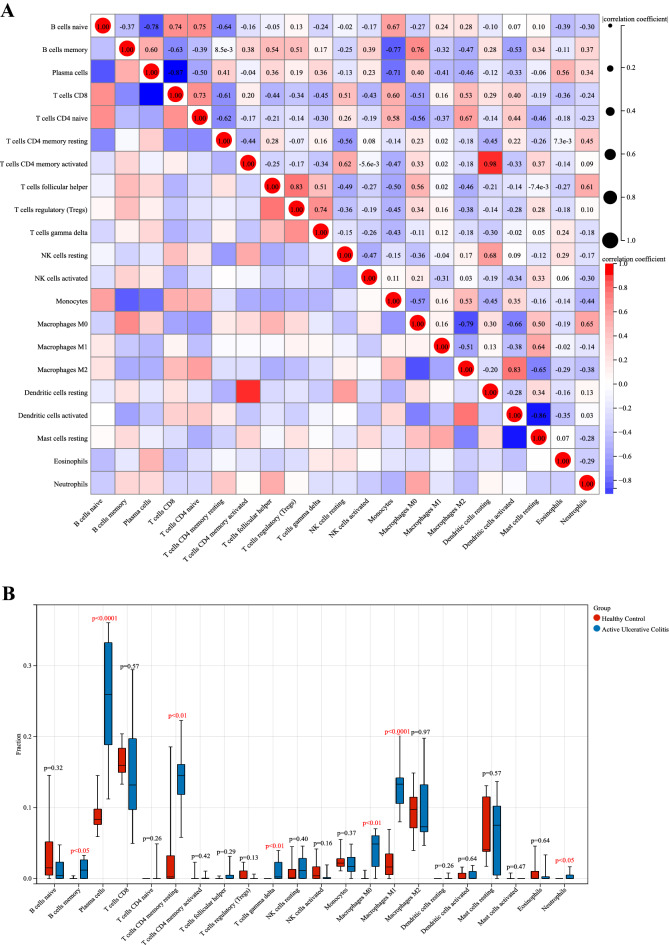
Correlation analysis and bar plot of differences among immune cells in the UC group. (**A**) Correlation analysis. Red indicates a positive correlation and blue indicates a negative correlation; the higher is the absolute value, the stronger is the correlation between immune cells. (**B**) Bar plot showing the proportion of each immune cell type between healthy and UC samples; red corresponds to healthy samples and blue to UC samples, *P* < 0.05.

A positive correlation was detected between naïve B cells and CD4 T cells (r = 0.75, *p* < 0.05), memory B cells and M0 macrophages (r = 0.76, *p* < 0.05), activated CD4 memory T cells and resting dendritic cells (r = 0.98, *p* < 0.01), follicular helper T cells and regulatory T cells (Tregs) (r = 0.83, *p* < 0.05), as well as M2 macrophages and activated dendritic cells (r = 0.83, *p* < 0.05). Instead, a negative correlation was detected between naïve B cells and plasma cells (r = − 0.78, *p* < 0.05), memory B cells and monocytes (r = − 0.77, *p* < 0.05), plasma cells and CD8 T cells (r = − 0.87, *p* < 0.05), M0 and M2 macrophages (r = − 0.79, *p* < 0.05), as well as activated dendritic cells and resting mast cells (r = − 0.86, *p* < 0.05) (Fig. 6A).

Differences in immune infiltrating cells between the intestinal tissues of healthy and UC patients were visualized by a bar plot, with statistically significant differences at *P* < 0.05. Plasma cells, memory B cells, resting CD4 memory T cells, γδ T cells, M0 macrophages, M1 macrophages, and neutrophils were all differentially elevated in the intestinal tissues of UC patients (Fig. 6B).

### Correlation between hub genes and immune-infiltrated cells in UC

**Figure 7:**
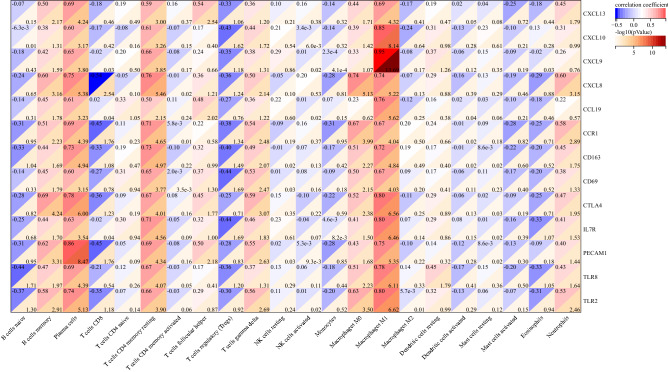
Correlation between hub genes and immune-infiltrated cells in UC. The darker is the red hue, the smaller is the *P* value.

The relationship between hub genes and immune-infiltrated cells in UC, which differed between UC and control samples, was evaluated by Pearson correlation (Fig. 7). M1 macrophages displayed a positive correlation with *CXCL13* (r = 0.69), *CXCL10* (r = 0.85), *CXCL9* (r = 0.95), *CXCL8* (r = 0.74), *CCL19* (r = 0.76), *CCR1* (r = 0.67), *CD163* (r = 0.72), *CD69* (r = 0.67), *CTLA4* (r = 0.80), *IL7R* (r = 0.80), *PECAM1* (r = 0.75), *TLR8* (r = 0.78) and *TLR2* (r = 0.80). Memory B cells exhibited a positive correlation with *CTLA4* (r = 0.78). Plasma cells showed a positive correlation with *PECAM1* (r = 0.86). M0 macrophages exhibited a positive correlation with *CXCL8* (r = 0.74) and *CCR1* (r = 0.67). Therefore, hub genes were strongly correlated with immune-infiltrated cells in UC.

## Discussion

UC originates from a disruption in the balance between the host's mucosal immunity and intestinal bacterial flora, resulting in an abnormal immune response to commensal non-pathogenic bacteria^6^. In the present study, gene expression data were obtained from the GEO database, which identified 174 upregulated and 32 downregulated DEGs in UC tissues. GO and KEGG functional classification indicated that DEGs were enriched mainly in immune response pathways, such as Toll-like receptor signaling, IL-17 signaling, and immune system process and chemokine signaling. Toll-like receptor signaling plays an important role in the pathogenesis of inflammatory bowel disease (IBD)^18^; whereas IL-17 signaling is involved in the development of colonic tissue damage and inflammation during UC^19^. CD4 T cells and natural killer (NK) T cells can promote the release of Th2-associated cytokines and Th17-associated pro-inflammatory cytokines, aggravating the UC intestinal inflammatory response^20,21^. Growing evidence points to the role of chemokine signaling in UC^22–24^. Overall, results of functional enrichment analysis suggested that immune factors played a critical role in UC pathogenesis.

UC occurs following dysregulation of the innate and adaptive immune responses to the intestinal microbiota in genetically susceptible hosts, with many different immune cell types involved^4,25,26^. Plasma cells, memory B cells, resting CD4 memory T cells, γδ T cells, M0 macrophages, M1 macrophages, and neutrophils were found to be more abundant in UC than healthy intestinal tissues. Plasma cells, B cells, and T helper cells are hallmarks of adaptive immune disorders associated with the onset and progression of UC intestinal inflammation^27–29^. Wang et al. suggested that antigens activated memory B cells, prompting them to differentiate toward plasma cells and produce antigen-specific IgG that initiated UC pathogenesis^30^. Hanai et al. suggested that leukocytapheresis acted therapeutically by reducing the ratio of CD4 + CD45RO + CD62L− effector memory T cells to CD4 + CD45RO + CD62L + central memory T cells in UC patients^31^. Earlier studies suggested that, in the presence of T cells, numerous types of immune cells might be triggered to produce more chemokines, followed by neutrophils infiltration into the colonic mucosa, thus directly and/or indirectly exacerbating the severity of UC-like chronic colitis^32^. γδ T cells expressing the Vδ2 chain produce IL-17 in the intestine of patients with long-standing IBD and are involved in the chronic inflammatory process^33^. Recent studies have shown that the global immune cell landscape of UC tissues is characterized by an increase in M0 macrophages and neutrophils^34^. Macrophages are key effector cells of the innate immune system and are crucial for intestinal mucosal stability^35,36^. In addition, they may serve as antigen-presenting cells and play a critical role in the initiation of the immune response^37^. Zhuang et al. highlighted the abnormalities of M1/M2 macrophage polarization at the onset and during development of UC^38^. Activation, migration, and degranulation of neutrophils are important mechanisms of intestinal injury in IBD^39^. Here, the identified UC-associated immune-infiltrated cells were involved in the progression of UC, as indicated by correlation between such cells and the colonic mucosal tissue of patients with UC. Nevertheless, the intricate network of interactions and regulation among immune cells means that more research is required to determine their exact role in UC.

According to the PPI network of DEGs, 13 out of 446 genes displayed an elevated degree of interaction and were upregulated in UC patients: *CXCL13*,* CXCL10*,* CXCL9*,* CXCL8*,* CCL19*,* CTLA4*,* CD69*,* CD163*,* CCR1*,* PECAM1*,* IL7R*,*TLR8* and *TLR2*. Chemokines can significantly increase chronic inflammation and intestinal tissue destruction in IBD through their ability to induce chemotaxis and leukocyte activation^40^. *CXCL8 (IL-8)* is an ELR + chemokine secreted by neutrophils, macrophages, and intestinal epithelial cells^41–43^. *CXCL8* has a strong chemotactic effect on neutrophils and activates their metabolism and degranulation^44^. In addition to being a feature of UC pathophysiology, neutrophils infiltration in the intestinal mucosa is also a functional indicator of adaptive immunity^45^. Both *CXCL9* and *CXCL10* belong to the family of ELR- chemokines, and target *CXCR3* as their receptor^46^. *CXCL9* is highly expressed in the intestinal mucosa of mice with experimental colitis and in UC patients (especially in lymphocytes, macrophages, and epithelial cells)^22^. *CXCL10* is a potent chemokine that is primarily secreted by monocytes and macrophages (including M1 macrophages) in IBD^47,48^. *CXCL9* and *CXCL10* recruit mostly Th1 cells, monocytes, and NK cells^49^. CXCL13 belongs to the ELR-chemokine family and targets CXCR5 as its receptor, and its mRNA expression levels are elevated in intestinal tissues of UC models^50^. In contrast to ELR + CXC chemokines, ELR- CXC chemokines lack chemotactic activity on neutrophils; instead, they are highly responsive to memory T cells and NK cells^50^. A humanized anti-*CCL21* monoclonal antibody has been identified as a potent marker for the diagnosis of active IBD and as a possible therapeutic agent for the prevention of IBD recurrence^51^. *CCL19* is involved in the progression of UC^52,53^. Given that *CCL19* induces the activation of MEK1-ERK1/2 and PI3K-AKT cascades in M1 macrophages^54^, *CCL19* is believed to exacerbate the progression of UC by inducing chemotaxis in M1 macrophages. *CCR1* is expressed on neutrophils, contributing significantly to tissue damage and mucosal dysfunction in UC^53,55^. In summary, the family of chemokines/chemokine-receptors was found here to correlate positively with M0/M1 macrophages and neutrophils. This indicates that immune-infiltrated cells and UC hub genes collectively influence UC through immune factors.

*CTLA4* serves as a negative regulator of the immune system, and is highly expressed on Tregs and activated T cells^56,57^. Currently, the level of *CTLA4* expression in UC remains unclear. Wang et al.^58^ and Magnusson et al.^59^ suggested that *CTLA4* expression in T cells was lower in UC patients than in healthy individuals. However, other studies have shown that *CTLA4* mRNA expression was significantly higher in colonic mucosal tissue from patients with active UC compared with controls devoid of inflammation^60^. Interestingly, in our study, *CTLA4* was upregulated in patients with UC and correlated positively with Tregs. Although Abatacept™ (*CTLA4*-Ig) has been effective against psoriasis^61^ and rheumatoid arthritis^62^ through its inhibitory effect on T cell activation, there is no evidence from a phase III clinical trial^63^ of its beneficial effect on UC. *CTLA4* may be upregulated in patients with UC via a feedback mechanism, but may fail to exert negative immune regulatory effects due to defects in its downstream pathways or reduced activation. Furthermore, blocking *CTLA4* or a Treg-specific reduction of *CTLA4* expression lead to increased numbers of plasma cells and memory B cells after vaccination^64^. This evidence points to the involvement of *CTLA4* in regulating the above immune cells, but whether it interacts and participates in the immune regulatory mechanisms of UC remains to be determined.

*CD69* is one of the first surface antigens expressed by T lymphocytes after activation, and its expression can act as a co-stimulatory signal to promote further activation and proliferation of T cells^65^. *CD69* is expressed on different leukocytes, including newly-activated lymphocytes, certain subtypes of memory T cells, infiltrating lymphocytes isolated from patients with chronic inflammatory disorders, Tregs, and NK cells^65–67^. The immunoregulatory function of *CD69* involves controlling the differentiation balance of Th/Treg cells and enhancing the suppressive activity of Tregs^68^. However, the exact mechanism of the interaction between *CD69* and UC requires further study. Soluble *CD163* is a specific macrophage activation marker that is reduced by anti-TNF-α antibody treatment in active inflammatory bowel disease^69^. Defining the landscape of mononuclear phagocytes in mesenteric lymph nodes provides evidence for the expansion of CD163 + Mono/MΦ-like cells in UC, highlighting the distinction between UC and CD^70^.

*PECAM1* (*CD31*) is expressed on the surface of endothelial cells, platelets, monocytes, neutrophils, T cell subsets, B cells, and dendritic cells, and has been reported also in plasma cells^71–73^. The interaction between endothelial cells and leukocytes is a key step in the inflammatory response, and *PECAM1* enables leukocytes to enter the site of inflammation and cause tissue damage^74^. Thus, *PECAM1* has an important role in inflammatory microcirculation injury. In the present study, *PECAM1* correlated positively with plasma cells, which suggested that plasma cells probably contributed to inflammation and tissue damage in UC through *PECAM1* expression. A further possibility is that *PECAM1* may promote plasma cells infiltration into inflammatory sites of the intestine in UC.

Interleukin-7 (IL-7) is a cytokine produced mainly by epithelial and stromal cells that regulates T lymphocyte homeostasis^75^. Almost all conventional mature T lymphocytes express high levels of IL-7 receptor (*IL-7R*), with naturally occurring Tregs being a special exception^76^. In healthy colon biopsies, intestinal epithelial cells produce IL-7 and mucosal T lymphocytes express *IL7R*^77^. A study has shown that genetic locus variants in the *IL7R* gene are associated with UC susceptibility^78^. In addition, elevated expression of IL-7 signaling pathway genes in blood CD8 + T cells at diagnosis was significantly associated with the course of IBD disease^78^.

*TLR2* upregulation in the gut of UC patients has been associated with increased antigenic stimulation of inflammatory and immune pathways activated by ligand binding^79^. Macrophages in the intestinal mucosa can rapidly engage in Toll-like receptor-mediated inflammatory responses to prevent pathogen invasion, but these innate immune responses can also trigger UC^80^. Documented increased expression of *TLR2* in macrophages from IBD patients^81^ corroborates our finding showing a positive correlation between *TLR2* and M0/M1 macrophages. Hence, we hypothesize that M0/M1 macrophages may mediate the inflammatory response to UC through *TLR2*. *TLR8* is a key component of innate and adaptive immunity, and it has been shown that its expression is increased in UC patients and that mRNA levels are positively correlated with the severity of intestinal inflammation as well as the severity of inflammation^75^.

**Figure 8:**
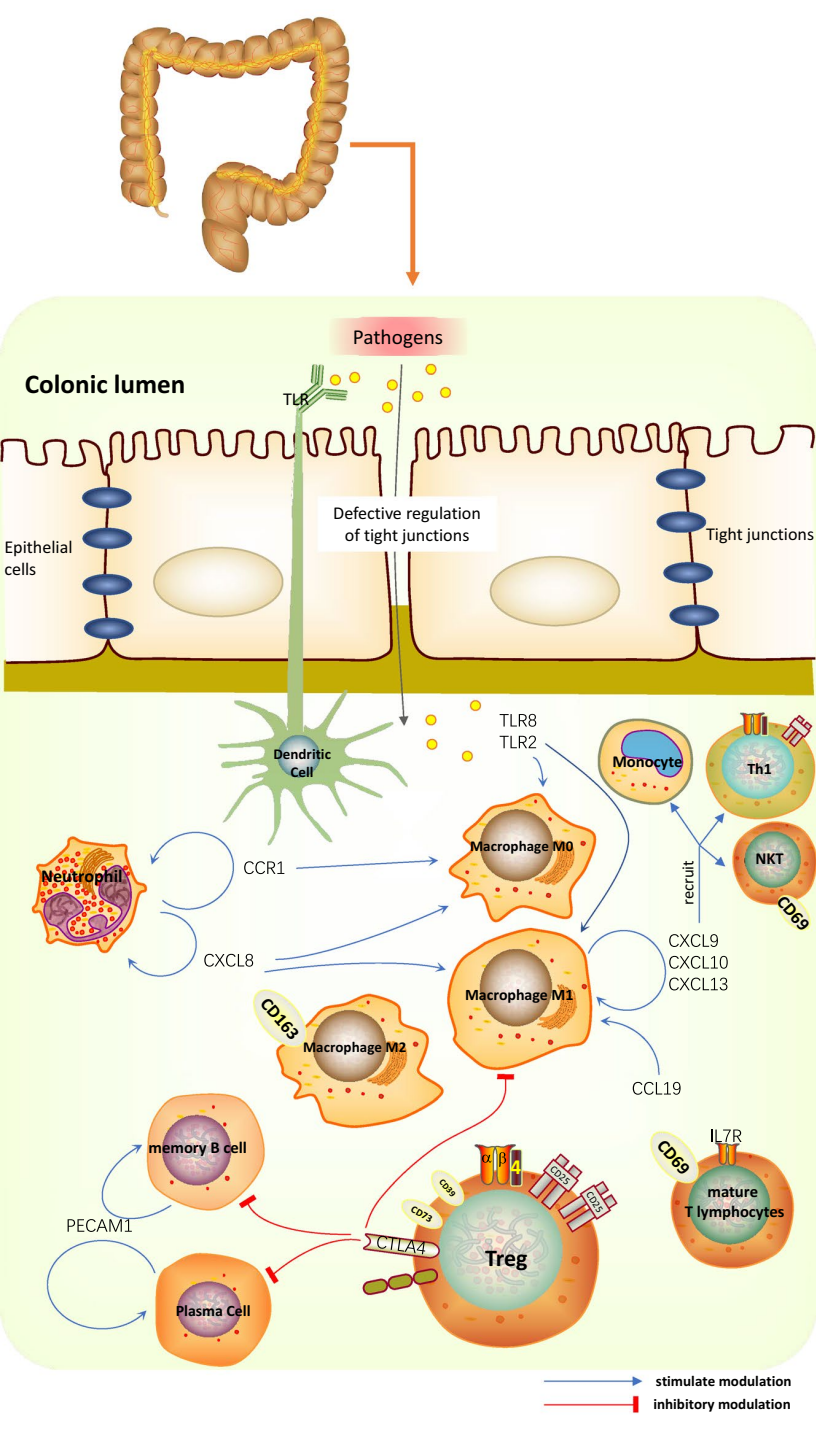
Hub genes and immune-infiltrated cells in UC.

We have mapped a proposed mechanism for the main results of this study (Fig. 8). Certainly, the above speculation requires further studies to verify the role of immune response in UC via the mutual regulation between hub genes and immune-infiltrated cells. The present study presented also some limitations. First, it was based on the GEO database, which is a secondary mining and analysis database of previously published datasets. Hence, the experimental results may differ from the conclusions of previous experiments, most likely due to biased data analysis caused by the small sample size. Second, the CIBERSORT deconvolution algorithm is based on limited genetic data, which may lead to inaccurate results due to different disease predisposing factors and the plasticity of disease phenotypes. Here, CIBERSORT was used to identify potential immune-related genes or immune infiltrating cells in UC. In addition, group centrality metrics^82^ are particularly useful because they describe the importance common to all hub genes and can be used to identification of hub genes by tools such as keyplayer^83,84^ (http://www.analytictech.com/keyplayer/keyplayer.htm) or Pyntacle^85^. Although we did not use other network tools or methods in this study, perhaps we will enrich our study with these tools for other studies in the future. Nevertheless, our study may still provide compelling evidence for further research on the potential of the identified immune-infiltrated cells or immune-related genes for the treatment and diagnosis of UC (Supplementary file).

## Conclusion

13 hub genes associated with immune-infiltrated cells in UC were identified in this study; they include *CXCL13*,* CXCL10*,* CXCL9*,* CXCL8*,* CCL19*,* CTLA4*,* CCR1*,* PECAM1*, *CD163*,* CD69*,* IL7R*, *TLR8* and *TLR2*. Additionally, bioinformatics analysis revealed M0/M1 macrophages, γδ T cells, plasma cells, memory B cells, and neutrophils to constitute the majority of immune-infiltrated cells in UC. These findings point to the immune response playing an important role in UC via mutual regulation between hub genes and immune-infiltrated cells.

## Supplementary Information

Supplementary Information.

## Data Availability

The datasets generated and/or analyzed during the current study are available in the [GEO] repository, [https://www.ncbi.nlm.nih.gov/geo/query/acc.cgi?acc=GSE65114].

## Abbreviations

UC: Ulcerative colitis
DEGs: Differentially expressed genes
GO: Gene Ontology
KEGG: Kyoto Encyclopedia of Genes and Genomes
GEO: Gene Expression Omnibus
STRING: Search Tool for the Retrieval of Interacting Genes
PPI: Protein-protein interaction
MCODE: Molecular Complex Detection
CXCL13: Chemokine (C-X-C motif) ligand 13
CXCL10: Chemokine (C-X-C motif) ligand 10
CXCL9: Chemokine (C-X-C motif) ligand 9
CXCL8: Chemokine (C-X-C motif) ligand 8
CCL19: C-C motif chemokine ligand 19
CTLA4: Cytotoxic T-lymphocyte associated protein 4
CCR1: C-C motif chemokine receptor 1
IL7R: Interleukin-7 receptor subunit alpha
TLR2: Toll-like receptor 2
TLR8: Toll-like receptor 8
PECAM1: Platelet and endothelial cell adhesion molecule 1
IBD: Inflammatory bowel disease
NK: Natural killer
